# Pathophysiology and management of reperfusion injury and hyperperfusion syndrome after carotid endarterectomy and carotid artery stenting

**DOI:** 10.1186/s13231-016-0021-2

**Published:** 2016-09-06

**Authors:** Muhammad U. Farooq, Christopher Goshgarian, Jiangyong Min, Philip B. Gorelick

**Affiliations:** 1 Division of Stroke and Vascular Neurology, Mercy Health Hauenstein Neurosciences, 200 Jefferson Street SE, Grand Rapids, MI 49503 USA; 2 Department Translational Science & Molecular Medicine, Michigan State University College of Human Medicine, Grand Rapids, MI USA

**Keywords:** Carotid endarterectomy, Carotid artery stenting, Cerebral blood flow, Cerebral hyperperfusion, Reperfusion injury, Risk factors and treatment of cerebral hyperperfusion syndrome

## Abstract

Cerebral hyperperfusion is a relatively rare syndrome with significant and potentially preventable clinical consequences. The pathophysiology of cerebral hyperperfusion syndrome (CHS) may involve dysregulation of the cerebral vascular system and hypertension, in the setting of increase in cerebral blood flow. The early recognition of CHS is important to prevent complications such as intracerebral hemorrhage. This review will focus on CHS following carotid endarterectomy and carotid artery stenting. We will discuss the typical clinical features of CHS, risk factors, pathophysiology, diagnostic modalities for detection, identification of patients at risk, and prevention and treatment. Although currently there are no specific guidelines for the management of CHS, identification of patients at risk for CHS and aggressive treatment of hypertension are recommended.

## Background

Cerebral hyperperfusion syndrome (CHS) is a relatively rare condition after carotid endarterectomy (CEA) or carotid artery stenting (CAS) but is potentially preventable. CHS may be defined as focal cerebral damage following a revascularization procedure, usually as a result of hyperperfusion. Bouri et al. suggest the following four criteria to define post-CEA CHS [1]:

1. Occurrence within 30 days post-CEA;
2. Clinic features such as new onset headache, seizure, hemiparesis, and glasgow coma scale (GCS) <15 or radiological features including cerebral edema or intracerebral hemorrhage (ICH);
3. Evidence of hyperperfusion (defined as a cerebral blood flow [CBF] >100 % or perioperative values) on imaging studies [e.g., transcranial doppler, single photon emission computerized tomography (SPECT) or magnetic resonance perfusion (MRP)] or systolic blood pressure >180 mmHg [2]; and
4. No evidence of new cerebral ischemia, postoperative carotid occlusion and metabolic or pharmacologic cause.

Hyperperfusion has been reported in 0.2 to 18.9 % of cases following CEA [2]. The incidence of CHS, however, is much less frequent. Moulakakis and colleagues in a retrospective review of 4689 patients undergoing CEA and 4446 patients undergoing CAS reported the incidence of CHS and ICH following CAS as 1.16 and 0.74 %, respectively, whereas following CEA the incidence of CHS and ICH was 1.9 and 0.37 % [3].

The term CHS has often been used interchangeably with cerebral reperfusion injury, and some authors argue that the latter term is more appropriate.

The pathophysiology of reperfusion related injury is not entirely clear, however, it is believed that dysregulation of the cerebral vascular system and hypertension resulting in an increase of CBF play a significant role.

Early recognition of CHS is important as it may be reversed at an early stage. Initially the edema associated with CHS is reversible, however, if CHS progresses to ICH the prognosis is not nearly as favorable as up to 30 % of patients remain at least partially disabled, and mortality rates are up to 50 % [4, 5].

This review will focus on CHS following CEA and CAS. Specifically, we will discuss the pathophysiology, diagnostic modalities for detection and identification of patients at risk, and prevention and treatment of CHS.

## Pathophysiology

The exact mechanism of CHS after CAS or CEA remains unclear but seems to be multi-factorial.

### Impairment of autoregulation

The normal brain has the ability to maintain constant intracranial pressure by an autoregulatory mechanism, when a change in blood flow occurs. Waltz in 1968 first described the impact of changes of systemic blood pressure on CBF in ischemic and non-ischemic cortex in a cat model of middle cerebral artery occlusion [6]. In non-ischemic cortex, cortical blood flow remained constant despite changes in systemic blood pressure, whereas in ischemic cortex, cortical blood flow varied directly with systemic blood pressure from 35 to 120 mm Hg [6]. Waltz hypothesized that cerebral ischemia produces an impairment of cerebral autoregulation [6].

Sundt and colleagues reviewed CBF in 1145 patients who underwent carotid endarterectomy from 1972 to 1981 [7]. They found that the most common causes of perioperative neurologic complications were intraoperative embolization and postoperative hyperperfusion phenomenon. Six patients had severe unilateral headache postoperatively and the mean increase in CBF was 40 ml/100 g/min. Mean increase in CBF for the five patients with ICH was 47 ml/100 g/min. Whereas mean increases in CBF for all other patients without these postoperative neurologic complications was 1 to 5 ml/100 g/min. They concluded that neurological complications including CHS are related to increase of cerebral perfusion after recanalization of the previous narrowed carotid artery [7].

Bernstein and colleagues described a 56-year-old man with a high-grade left carotid stenosis who had left CEA [8]. The patient developed a severe left-sided headache on the first postoperative day. On the second postoperative day he had grand mal seizures. He had right hemiparesis and died of a left ICH on the sixth postsurgical day. At postmortem examination, the small arteries and arterioles of the left cerebral cortex showed reactive edema and hyperplasia of endothelial cells, extravasation of erythrocytes, and fibrinoid necrosis [8]. These features of altered vascular pathology are similar to those seen in the brain with malignant hypertension. They surmised that chronic cerebral ischemia distal to high-grade carotid artery stenosis led to chronic vasodilatation, loss of autoregulation, and a consequent absence of arterial vasoconstriction to protect the capillary bed [8].

Postoperative hypertension in the setting of impaired autoregulation may cause an increase in cerebral perfusion pressure (CPP) as CPP is dependent on mean arterial pressure. Acute increase in CPP in areas of infarcted/necrotic or hypoperfused tissue can lead to ICH. This may be more pronounced when there is impaired vasoreactivity of cerebral vasculature and inability of arterioles to constrict in the setting of increased perfusion resulting in ICH [9].

### Chronic hypertension, microangiopathy and blood brain barrier damage

In general, patients with severe carotid artery stenosis often have underlying systemic hypertension and undergo CEA to reduce the risk of stroke in a vascular bed that may be subject to chronic cerebral ischemia. Baseline chronic hypertension might play a major role in the development of CHS. Preoperative longstanding hypertension leads to endothelial dysfunction and microangiopathy which can result in a breakdown of the blood brain barrier (BBB). Breakdown of the BBB was observed previously in an animal model of cerebral hyperperfusion [10]. There is evidence of extravasation of serum albumin after BBB breakdown in animal experiments showing that there is activation of the transforming growth factor beta (TGFβ) signaling pathway. This results in the induction of cerebral edema and seizure-like activity in patients with no prior edema [11]. Damaging the BBB allows extravasation of toxins and edema into the brain parenchyma. Additionally, underlying lipohyalinosis in the setting of hypertension can lead to the development of Charcot–Bouchard aneurysms and the subsequent occurrence of ICH [9].

### Role of nitric oxide and free radicals in impairment of autoregulation and BBB dysfunction

A presumed mediator of impairment of autoregulation and dysfunction of BBB in CHS is nitric oxide, which leads to vasodilatation and increases in cerebral vessel permeability [12]. Dohare and colleagues demonstrated that high levels of nitric oxide generated by nitric oxide synthase isoforms are responsible, at least in part, for exacerbating the neuronal damage in the rat model of middle cerebral artery ischemia/reperfusion with an intraluminal filament [13]. Increased nitric oxide expression and free radical production may last up to 48 h after reperfusion. It has been shown that oxygen-derived free radicals associated with CEA play a role in furthering ischemic injury even after short-term carotid artery clamping [14, 15].

### Baroreceptor dysfunction

Baroreceptor dysfunction is also known as baroreflex failure syndrome (BFS). This can be related to baroreceptor denervation after bilateral CEA and may lead to CHS. Baroreceptor dysfunction can also cause a progressive increase in blood pressure after CEA which is challenging to control even with blood pressure lowering therapy. Fluctuations in blood pressure after CEA can last up to 12 weeks due to baroreceptor denervation. One study showed that 6.6 % of patients with bilateral CEA developed CHS as compared to 1.1 % of patients with unilateral CEA. Therefore, contralateral CEA performed within 3 months of CEA on the other side increases the risk of CHS [16–18].

Sometimes, there is carotid baroreceptor stimulation during a carotid artery endovascular procedure via a balloon or carotid stent manipulation. The stimulation results in transient bradycardia and hypotension leading to cerebral ischemia. In some situations, the stimulation can be quite prolonged even more than that seen in patients with CEA during clamping of the internal carotid artery. These patients are at risk of CHS due to rebound arterial hypertension and also due to cerebral ischemia that occurs during the procedure [3, 19].

Key points about the pathophysiology of CHS are listed in Table 1.

**Table 1:** Key factors in the pathophysiology of cerebral hyperperfusion syndrome [1, 2, 12–23]

Factor	Pathophysiology
Impaired auto-regulation and baroreceptor dysfunction	Fluctuations in blood pressure Post-operative hypertension Increase in cerebral perfusion pressure Risk of intracerebral hemorrhage in hypo-perfused tissues Transient bradycardia and changes in cerebral blood flow
Chronic hypertension, microangiopathy and blood brain barrier	Endothelial dysfunction and microangiopathy Increased vessel permeability Breakdown of blood brain barrier Extravasation of albumin Activation of TGFβ signaling pathways Release of nitric oxide
Formation of free radicals	Lipid peroxidation Vascular endothelial damage Cerebral edema
Degree of chronic carotid stenosis	Chronic hypoperfusion Endothelial damage Imbalance of vasodilatory chemicals
Collateral circulation	Changes in cerebral blood flow Cerebral vasoreactivity

## Imaging modalities used in the prediction and diagnosis of cerebral hyperperfusion syndrome

There are several imaging modalities and techniques used to investigate patients for CHS. These include but are not limited to transcranial doppler (TCD), computerized tomography (CT), magnetic resonance imaging (MRI), MR perfusion (MRP) and single-photon-emission CT (SPECT).

### Transcranial doppler

TCD is the most commonly and widely available technique that can be used for the evaluation and prediction of the risk of CHS in preoperative, perioperative and postoperative phases. The main advantage of TCD is that it is non-invasive and provides real-time information. One may observe preoperative cerebral hypoperfusion and postoperative cerebral hyperperfusion. Also, TCD detects cerebral embolic signals that may lead to ischemia [2, 3].

TCD measures CBF velocity in the middle cerebral artery (MCA) and can be of value in predicting a difference in CBF in patients with CHS. Auto-regulation has no effect on the diameter of the MCA. Therefore, changes in MCA flow velocity correlate nicely with changes in MCA perfusion. If there is a significant reduction in CBF velocity of intracranial blood vessels in the preoperative phase as compared to baseline values, it will be associated with postoperative hyperperfusion. On the other hand, a 1.5-fold postoperative increase of MCA mean flow velocity compared with preoperative levels may predict the occurrence of CHS [18, 20].

Other TCD criteria for the prediction of postoperative hyperperfusion in patients with recent CEA have been defined. These include low perioperative distal carotid artery pressure (<40 mmHg), and an increase in peak blood flow velocity and pulsatility index of >100 % after declamping of the carotid artery in CEA [18, 21, 22].

Moreover, cerebral vasoreactivity can predict the risk of CHS, and this can be measured using TCD. In a normal person, the administration of carbon dioxide or acetazolamide will lead to a rapid increase in CBF ranging from 20 to 80 % due to dilatation of cerebral blood vessels. In patients with chronic cerebral ischemia, the cerebral blood vessels are already maximally dilated, and there is no significant change in CBF after the administration of carbon dioxide or acetazolamide. This is called low or impaired cerebral hemodynamic reserve. Using TCD, patients with low preoperative cerebrovascular reserve are at risk of developing cerebral hyperperfusion and CHS [18, 23, 24].

Also, it has been shown that TCD-derived low intraoperative distal internal carotid artery pressure (d ICAP) (<40 mmHg) has a high predictive value for postoperative hyperperfusion and CHS [21]. Moreover, a significant increase in mean internal carotid artery volume flow (MICAVF) has been reported in patients with CHS during the symptomatic period [3, 17].

The limitations of TCD should be kept in mind, and these include but are not limited to various technical problems such as an insufficient cranial window for insonation, variations of intracranial blood vessels forming the circle of Willis making insonation difficult, and cervical vessels which can affect velocities of intracranial vessels.

### CT

**Fig. 1:**
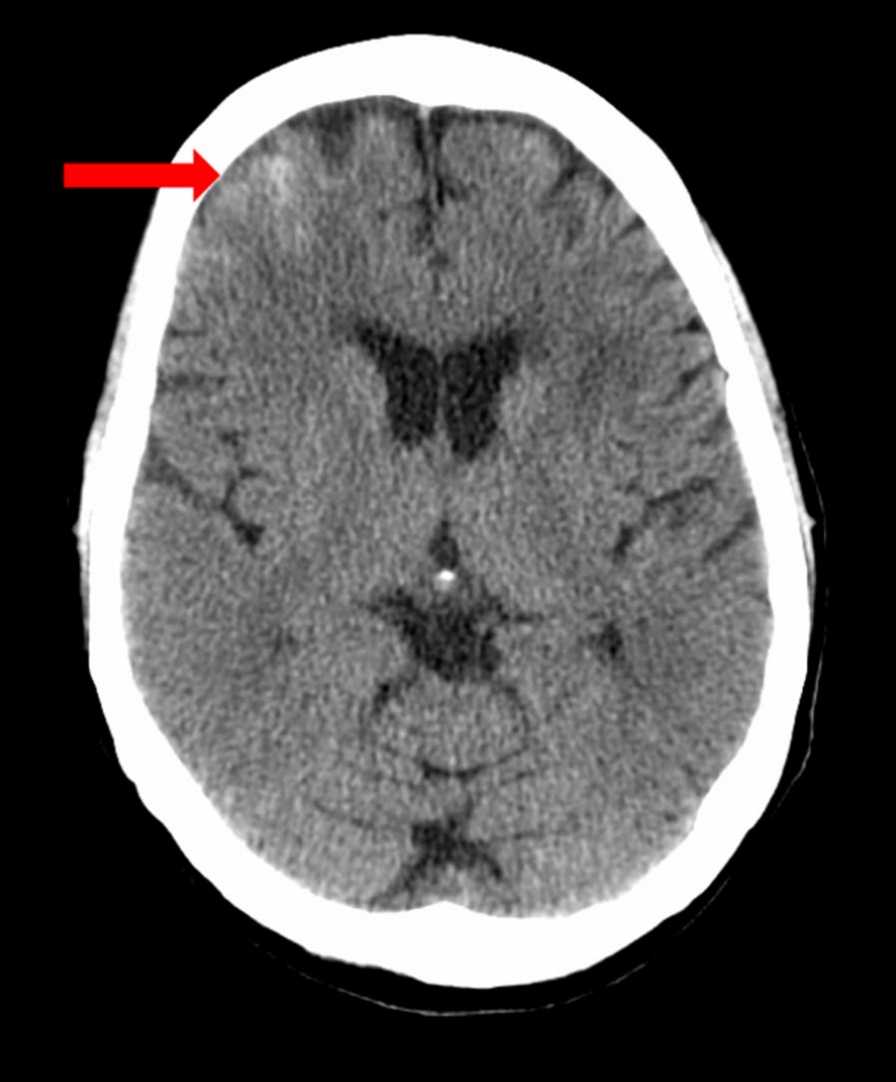
CT scan of the brain (axial sequence) shows an area of hyperdensity in the right frontal lobe suggestive of intracerebral hemorrhage (*arrow*) in a 67 year old woman who underwent right carotid endarterectomy (CEA) for the treatment of a 95 %* right* ICA stenosis. The patient post-operatively developed headache, photophobia and intermittent dizziness. This CT brain was done almost 24 h after the CEA. Systolic blood pressure was in the 170 s mm Hg and difficult to control as after CEA there was thought to be a clamp injury to the right carotid artery bulb. However, the patient did well clinically and at her 3 month follow up office visit, she had no residual neurological deficits

CT of the brain completed right after CEA can be completely normal in patients with CHS. Later on, findings develop which may include diffuse cerebral edema, patchy white matter changes, mass effect, and intracerebral hemorrhage (Fig. 1). These changes are sometimes more marked in the posterior circulation involving posterior parietal-occipital regions as there is a lack of sympathetic innervation in the posterior circulation of the brain. Overall, however, CT brain before or after CEA is of limited value for the evaluation of CHS as such findings can be non-specific. Thus, CT brain is not a useful tool for prediction of CHS [2, 3].

### MRI

**Fig. 2:**
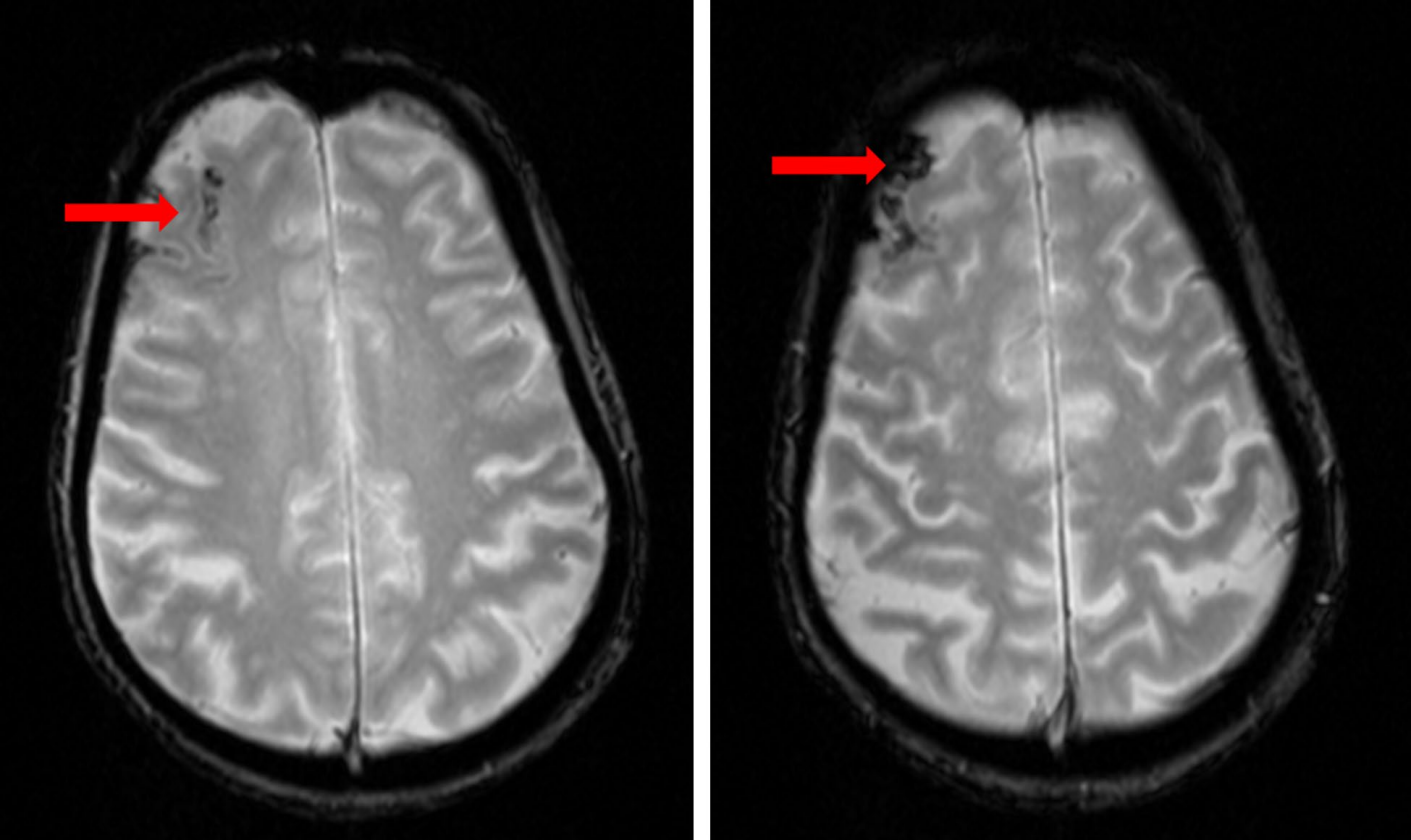
MRI of the brain (axial sequence) gradient recall echo (GRE) image shows hypointense foci in the right frontal region consistent with hemorrhage (*arrows*) in the same patient mentioned in Fig. 1

MRI of the brain is more sensitive to detect ischemic changes but not necessarily to predict the risk of CHS. MRI abnormalities can be similar to those seen on CT of the brain including white matter edema, focal infarction or hemorrhage (Fig. 2). However, in many cases of CHS, MRI of the brain may be unremarkable [2, 3].

### MR perfusion

MR perfusion scan can show a difference in CBF in the cerebral hemispheres. Measurement of preoperative CBF by using perfusion-weighted imaging can help identify patients at risk of cerebral hyperperfusion after CEA in the absence of contralateral internal carotid artery stenosis. Fukuda et al. showed a significant correlation between preoperative CBF and increase in CBF immediately after CEA (p < 0.0001). They studied the relationship between hyperperfusion immediately after CEA and elevated preoperative cerebral blood volume and noted that no patient with normal preoperative cerebral blood volume showed post-CEA hyperperfusion. Furthermore, elevated preoperative cerebral blood volume was the only significant independent predictor of post-CEA hyperperfusion [2, 3, 25].

### Single-photon-emission CT (SPECT)

Single-photon-emission CT of the brain is a sensitive method to identify patients at risk of hyperperfusion after CEA and for recognizing CHS. It helps to differentiate between brain ischemia and hyperperfusion. Similar to TCD, it can help determine CBF reserve after carbon dioxide or acetazolamide administration and also detect postoperative cerebral hyperperfusion, especially if hyperperfusion persists between the 1st and 3rd postoperative day. Ogasawara et al. showed that intraoperative MCA blood flow velocity monitoring by using TCD was a less reliable method to detect cerebral hyperperfusion after CEA than postoperative MCA blood flow velocity monitoring by SPECT. In this study, TCD monitoring could not be completed in one out of seven patients diagnosed with post-CEA hyperperfusion on SPECT imaging and who later developed CHS. SPECT technique is superior to TCD when there is occlusion or hemodynamically significant stenosis of the ipsilateral MCA [26–28].

Features of key imaging modalities in the diagnosis of CHS are listed in Table 2.

**Table 2:** Key imaging modalities used in the diagnosis of cerebral hyperperfusion syndrome [1–3, 9, 26–32]

Imaging modality	Key role and findings on imaging studies
Transcranial doppler	Non-invasive and provides real time information Detect cerebral embolic signals during CEA Accurately measures cerebral blood flow velocity Measurement of cerebral vasoreactivity Determination of distal internal carotid artery pressure Changes in mean internal carotid artery volume flow
CT and MRI brain	Detection of : Ischemic changes and focal infarction White matter edema Intracerebral hemorrhage
MR perfusion	Measurement of cerebral blood flow/volume and inter-hemispheric differences Contralateral carotid artery stenosis and flow difference
SPECT of the brain	Cerebral blood flow measurement CO2 and acetazolamide studies to determine flow changes Accurate measurement of middle cerebral artery blood flow velocity

## Prevention and treatment of cerebral hyperperfusion syndrome

Preventive strategies for CHS include proper blood pressure control in the perioperative period, and consideration of timing of surgery, type of anesthesia, and use of free radical scavengers. These strategies are discussed below.

### Blood pressure control

An important factor in the prevention of CHS is proper control of blood pressure. It may be prudent to carefully monitor blood pressure in patients with increased CBF as hypertension may subsequently develop postoperatively even in normotensive patients. Blood pressure control in patients with CHS may be challenging, and even when there is normal blood pressure, CHS can occur. Therefore, whereas it is recommended to have careful control of blood pressure, there are no definitive guidelines about the target blood pressure in these patients and for how long blood pressure needs to be controlled [2, 18, 21, 29, 30].

One should be cautious when selecting a drug for blood pressure control in these patients as some of the anti-hypertensives with vasodilatory effects may worsen the outcome. Medications in the latter group include calcium channel blockers, sodium nitroprusside, glycerol trinitrate, and angiotensin II inhibitors. Beta-blockers reduce arterial blood pressure and have little effect on the intracranial pressure within the autoregulatory range. Therefore, beta-blockers potentially can be used to treat hypertension in patients with brain injury. A limitation to some of the early generation beta-blockers is that they may be associated with blood pressure variability. However, other medications which may be more suitable for these patients include mixed alpha-adrenergic antagonists and beta-adrenergic antagonists such as labetalol. These medications help reduce cerebral perfusion pressure and mean arterial pressure without any direct effect on CBF. Patients with CHS may have elevated catecholamine concentrations. Therefore, medications like clonidine, a centrally acting sympatholytic, may be useful. Blood pressure in patients with CHS should be controlled with either labetalol or clonidine, which do not increase CBF [2, 3, 19, 21].

### Timing of carotid endarterectomy

According to the American Heart Association and American Stroke Association Guidelines, the benefit of CEA is greatest if it is done within 2 weeks of the ischemic stroke or transient ischemic attack [31]. However, there is a potential risk of CHS and cerebral hemorrhage if surgery is done early in patients with large cerebral infarction or stroke in evolution. Moreover, in the case of bilateral carotid stenosis, the risk of CHS is higher in a patient who undergoes CEA in less than 3 months of the initial procedure on the contralateral side. These factors should be taken into account when planning CEA [17, 32].

### Type of anesthetic

Some of the general anesthetics may lead to cerebral hyperperfusion and increase the risk of CHS. Therefore, one should be careful when selecting an anesthetic and its dose as high doses of a volatile halogenated hydrocarbon anesthetic may lead to the development of CHS. Some anesthetics such as isoflurane are favorable when used in patients with cerebral injury as these have a vasodilatory effect on cerebral blood vessels. However, the effects of isoflurane on the cerebral metabolic rate and cerebral autoregulation are dose dependent, and this should be kept in mind as higher doses can potentially lead to CHS. Similarly, nitrous oxide is a safe anesthetic at a concentration of less than 70 % and has no significant effect on cerebral autoregulation. A combination of anesthetics may lead to complications. For example, nitrous oxide combined with isoflurane may cause cerebral vasodilation. Other anesthetics, such as propofol, normalize CBF which may result from its effect on cerebral metabolism, and thus, it can potentially be used in patients with CHS [3, 33–35].

### Use of anti-epileptic medications

There is no indication for prophylactic use of anti-epileptic drugs in patients with CHS. However, if a patient has periodic lateralized epileptiform discharges on EEG or a clinical seizure spell, treatment with an anti-epileptic drug may be indicated [2, 3, 36].

### Pre-treatment with free radical scavengers

As mentioned above, oxygen-derived free radicals can lead to brain injury in patients with CHS. Free radical scavengers such as edaravone may minimize this damage. Edaravone inhibits lipid peroxidation and vascular endothelial damage. In one case series, use of edaravone helped to reduce the incidence of CHS after CEA as measured by SPECT [37]. The evidence for the use of antioxidants and free radical scavengers is limited, and no definite recommendation about their use in the clinical practice can be made at this time.

### Miscellaneous agents: role of mannitol, hypertonic saline, and other treatments

In patients with cerebral edema, mannitol and hypertonic saline may be used. However, the evidence for the use and potential beneficial effect of these medications in patients with CHS and their effect on long-term prognosis is not clear. Other treatments such as corticosteroids and barbiturates have been administered in select patients. In patients with cerebral edema, adequate sedation and hyperventilation may also be effective [3, 26, 28, 38]. Similar to what was mentioned in the prior section about antioxidants, no definite recommendation can be made about the use of mannitol, hypertonic saline, corticosteroids and barbiturates in patients with CHS.

Strategies for the prevention and treatment of CHS are listed in Table 3.

**Table 3:** Key factors in the prevention and treatment of cerebral hyperperfusion syndrome [1–3, 9, 22, 23, 26, 32, 34, 38]

Treatment modality	Comment
Blood pressure control	Strict control of blood pressure is recommended Lower blood pressure even in normotensive patients There are no definite guidelines about blood pressure parameters and management should be individualized Avoid medications which have vasodilatory effect such as calcium channel blockers Labetalol and clonidine are better options to treat elevated blood pressure in these patients
Timing of carotid surgery	Carotid endarterectomy or stenting should be done within 2 weeks of transient ischemic attack or stroke Patient is at risk of cerebral hyperperfusion syndrome if they underwent contralateral carotid endarterectomy in past 3 months
Type of anesthetic	High doses of volatile halogenated hydrocarbon anesthetics may lead to cerebral hyperperfusion syndrome Isoflurane is safer to use in these patients but can cause complications at higher doses Nitrous oxide is also safe but should not be used with isoflurane Propofol normalizes cerebral blood flow and is a safe option
Use of anti-epileptic medications	Prophylactic use of an anti-epileptic drug is not recommended If patient has lateralized epileptiform discharges or a clinically manifest seizure spell, an anti-epileptic drug may be administered
Use of hypertonic saline and mannitol	The evidence about the use of hypertonic saline and mannitol is not strong but may be administered if the patient has cerebral edema Corticosteroids and barbiturates are not indicated in most cases Hyperventilation and sedation may be administered if the patient has cerebral edema

## Conclusion

CHS is a potentially life-threatening complication of CEA and carotid stenting. It can happen within a few days postoperatively, but presentation can be delayed for weeks. Timely diagnosis and treatment are necessary. If these patients are treated early, they may do better as there may be minimal complications and a good recovery. However, with a delay in diagnosis, the mortality rate may be up to 50 %, especially in those patients who develop ICH. Patients with CHS may develop headache, focal neurological deficits, cerebral edema, brain hemorrhage and seizures. Most of the patients have hyperperfusion. However, a minimal to moderate increase in brain perfusion can lead to CHS.

Clinicians should be aware of the main putative risks for CHS such as high-grade carotid artery stenosis, poor collateral blood flow, post-operative hypertension and hyperperfusion, impaired cerebrovascular reserve, intra-operative distal carotid pressure of <40 mmHg, intraoperative ischemia, and recent contralateral CEA. TCD is the most commonly and widely available technique used in the perioperative period to monitor for cerebral hyperperfusion. Control of blood pressure with labetalol and clonidine may be useful for the prevention and treatment of CHS [2, 3, 18].

## Abbreviations

CHS: cerebral hyperperfusion syndrome
CEA: carotid endarterectomy
CAS: carotid artery stenting
CBF: cerebral blood flow
GCS: glasgow coma scale
ICH: intracerebral hemorrhage
MRP: magnetic resonance perfusion
SPECT: single photon emission computerized tomography
TCD: transcranial doppler
